# Leveraging enhanced perceptual markings for accident prevention in long-range applications near freeway exits: Evidence from driving simulation experiments

**DOI:** 10.1371/journal.pone.0331345

**Published:** 2025-10-15

**Authors:** Jingjing Zhang, Naikan Ding, Shuhao Wu, Guangyao Huang, Linsheng Lu

**Affiliations:** 1 Guangxi Transportation Science and Technology Group Co., LTD, Nanning, China; 2 Intelligent Transportation Systems Research Center, Wuhan University of Technology, Wuhan, China; 3 Engineering Research Center of Transportation Information and Safety, Ministry of Education, Wuhan, China; 4 Shaoguan Research Institute of Wuhan University of Technology, Shaoguan, China; Southwest Jiaotong University, CHINA

## Abstract

Perceptual markings are extensively applied in practice for accident prevention, especially at the high-risk segments and/or sites, such as the freeway exits. However, most of the present perceptual markings are tested for a short-range of segment, which could hardly guide its practical application in real-world scenarios, such as the freeway exits. The long-range application and performance of them are barely rigorously investigated in previous research, and how to effectively accommodate the cost-effective perceptual markings in the long-range application is unaddressed. Given this, we proposed a novel perceptual marking form, i.e., enhanced linear perspective markings (ERLPMs) in fixed-angle or variable-angle patterns, by add appropriate gaps between adjacent marking groups, to accommodate the long-range application on freeways. A series of driving simulation experiments were conducted to test the performance of the ERPLMs on adjusting drivers’ speed, acceleration, distance and time headways. The results show that 1) the ERLPMs effectively led to significant reduction of speed and increase in headways while vehicles approaching the exits in a long-range segment; 2) the greatest speed reduction (0.40 m/s), acceleration reduction (0.22 m/s^2^), distance headway increase (10.75 m), and time headway increase (0.41 s) were observed as compared with the baseline (no extra markings); and 3) the fixed-angle pattern with θ=120∘ had the most impressive and stabilized performance on the speed and headway control aspects. The findings of the study suggest that the ERLPMs are capable of adjusting driver behaviors smoothly and bring substantial benefits for accident prevention at freeway exit area, which could be a support for the practical usage of various markings in the long-range on freeways.

## 1 Introduction

### 1.1 Perceptual markings as a measure for accident prevention

Crashes on freeways usually lead to deaths and severe inuries, expecially at the diverging area while vehicles approaching exits. It was reported that approximately 30% of freeway accidents occur at the exit area, and the accident rate at exits is even twice as high as that at entrances [1,2]. The situation is even worse in low- and middle-income countries, where large traffic volume, highly mixed traffic, overspeeding and other violative and/or illegal driving issue, less effective traffic control measures are not rare to see and are extremely contributive to crashes on freeways.

To addresss this long-standing and threatening issue, especially in the low- and middle-income countries, the effective, low-cost, and readily applicable countermeasures for accident prevention at freeway exits are urgently needed. Over the past decades, road perceptual markings (also known as “perceptual treatments” or “perceptual countermeasures”) are representative and prevailing ones. Some of the pionners in this area, such as, [3,4,5], Reinoud J. Bootsma [6], Alfonso [7,8] 2022), Xiaohua Zhao [9] 2023, 2015, [10,11]), and so on, have long argued the fundamental role of perceptual markings on road surface as a effective visual guidance by affording drivers necessary visual cues to facilitate their speed control, steering on curves, and crash risk mitigation. In previous studies, a variety of forms and patterns of perceptual markings wasdesigned, tested, and validated on highways, rural roads, and urban expressways, with well-acceptable driving behavior and crash risk intervention effects. Conventionally, percetual markings are used as an accident prevention measure by reducing speed. For examples, Montella et al. [7] used peripheral transverse line markings to mitigate crash risk on a tangent segment of a rural road with a major 5.46 m/s reduction in speed. Calvi [12] investigated the speed reduction performance of a series of perceptual treatments on a curve segment of a rural road, and found a 6km/h speed reduction in maximum produced by the red peripheral transverse markings. Zhao et al. [10] evaluated the effects of longitudinal speed reduction markings on left-turn direct connectors. Wood and Donnell [13] reported impressive 34.8% and 30.7% reductions in total crashes, fatal and injury crashes at a suburban highway exits area after implementing of a text-based warning pavement marking. [14,14,15] proposed innovative perceptual markings based on drivers’ visual perception theories and paved them on real-world freeways, and found the maximum speed reductions of 1.139 m/s with regular longitudinal markings, 0.972 m/s with a kind of reverse linear perspective markings, 1.108m/s with a kind of peripheral transverse line markings.

### 1.2 The dilemma of long-range application of perceptual markings

Indeed, the above perceptual markings had impressive speed reduction effects and could be expected with substantial accident prevention and crash risk mitigation performance, but nearly all of them were installed within a short range, especially on real-world freeways (nornally less than 300m), which could hardly effectively guide the practically application of the perceptual markings in varying natural scenarios, such as the freeway exits. Specifically, the long-range installation of the perceptual markings on freeways, especially at the accident-prone sites (exits), is still an open issue to be addressed. The practical application of these perceptual markings in a long range to accommodate the specific traffic flow and driver behaviors at accident-prone sites remains a major challenge, and the performance of these markings in a long distance is rarely investigated by far.

Actually, the long-range application issue could be a struggling and frustrative dilemma the perceptual markings faced with. On the one hand, the perceptual markings should be installed along the accident-prone or high-risk segments, which usually last for a couple of kilometers or more, to produce the enhanced accident prevention performance. So, intuitively, it seems that simply installing these perceptual markings in a long range along the accident-prone or high-risk segments on freeways could be fair enough. However, there could be two drawbacks. First, more investments are needed for a long-range installation, which is actually contradictory to the “cost-effective” nature of the perceptual markings. Second, an overdense installation of the perceptual markings on road may give rise to unwelcome and unexpected negative effects on traffic safety due to a sudden high visual load to drivers passing by [16,17,18]. Particularly, according to our previous findings [14,19,15], the markings installed in a 2m unit (one meter marking and one meter gap) could largely be the most appropriate pattern producing applaudable speed reduction and crash risk mitigation effects and without leading to unbearrable visual load. Accordingly, it seems that the perceptual markings are not appropriate to be installed consecutively in an overmuch long segment (usually 2km or more) on freeways. On the other hand, according to the visual perception and cognitive psychology theories [20,21], if the perceptual markings are only installed in a quite limited range (around 500m as in most previous studies), the speed reduction and/or any other behavioral intervention and crash risk mitigation could be really negligible to the local traffic flow within its influence area. Because the drivers need enough and continuous stimuli from the visual field with the markings to enhanced their speed perception and distance perception so as to possibly lead to stabilized and substantial speed reduction and headway control effects [22]. Obviously, the ideal solution to the dilemma is to install the perceptual markings in a long rang without sacrificing the costs and bring adversial visual load to drivers, which, to our best knowledge, is absent in the literature of the research of perceptual treatments on roads by far.

### 1.3 The compatibility issue of speed and headway interventions of perceptual markings

In addition to the long-range application issue, the previous perceptual treatments were predominantly only designed and tested for speed reduction, little is known concerning their influence on headway adjustment performance. It is well believed that any safety benefits of the perceptual markings can hardly be the result of speed reduction alone [23,24,25,26]. A comprehensive longitudinal and lateral driving behavioral adjustment is needed to gurantee substantial and stabilized safety improvement [27,28,29]. Speed reduction alone was even questioned as an approporiate indicator to measure safety performance [30]. Moreover, the mere focus on speed reduction markings and speed reduction effect seemly did not well highlight the usefulness of perceptual markings on accident prevention. Actually, based on the fundamental visual perception theories, where the “perceptual” markings rooted from, and our previous empirical field test, observations, and findings, the functional design domain of the markings can be well extended to headway control aside from speed reduction under the premiss of well design of the markings according to visual perception theories [20,31,32].

Previously, for the purpose of obtain a better speed reduction and/or accident prevention performance, researchers and traffic engineers paid a great deal of efforts on the shape, color, and spatial layout of the markings. Generally, the longitudinal [3,33,34,10,14], transverse (or named as “herringbones”) [3,7,8,12,35,15], and chevron [36] ones are the most commonly seen shapes of markings. Garach et al. [37] specifically examined the width of longitudinal markings on driving speed perception. White, yellow, red, and green are the most adopted colors as among the percetual markings in the above, to expect to easily catch drivers’ attention and to be a more effective visual stimuli and guidance. [14,38,15], 2021a, 2021b) particularly focused on the spatial layout of the markings, and verified that the longitudinal gap between two adjacent markings played an important role in drivers’ speed and distance perception, and eventually adjusted driving behaviors. The cornerstone underpinning the above visual elements (shape, color, and spatial layout) as a possible solution for enhanced perceptual markings is actually the human visual perception mechanism of speed and distance, which relies on various visual cues inherent in optical flows due to relative motion between the drivers (eyes) and their surroudings [20,39,40,41,42]. Specifically, the temporal and spatial patterns of the markings, with regard to the drivers’ eyes, were understood to produce the visually temporal and spatial frequencies and variations, which stimulate visual cues for spatial and motion interpretation. The temporal frequency is also widely known as the “edge rate”, which was defined as the rate at which local discontinuities cross a fixed point of reference in the observer’s field of view, or could be explained as the number of markings passing by within a time unit, and accordingly be measured by the unit of Hz. [38,38] have revealed that on freeways with a general speed limit of 100km/h, when the edge rage fell in [5Hz, 12Hz] (the gap between two adjacent markings was 2m), the edge rate could generate the strongest illusion of ego-speed overestimation, which propelled drivers decelerate spontaneously. Similarly, “the discontinuity effects [42]” and visual linear perspective effects [19,43] were verified to be the predominant visual cues leading to distance underestimation of drivers, which were explained as that the discontinuious visual elements (i.e., the markings) on ground distrupt the orginally well-founded visual perception system.

**Fig 1:**
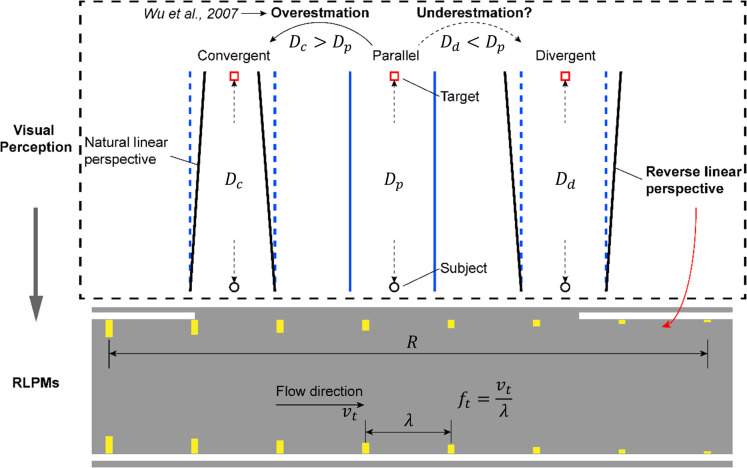
The reverse linear perspective and RLPMs (originally extracted from [43]).

By integrating the above-mentioned ego-speed overestimation and distance underestimation effects, we previously proposed a kind of unique transverse perceptual markings, i.e., the reverse linear perspective markings (RLPMs) (see Fig 1, [19,43]. The performance of RLPMs was verified in a short range of segments on freeways that they presented advantages on both speed reduction, headway control, and crash risk mitigation as compared with its counterparts of other regular perceptual markings.

Yet, when it comes to long-range application of the perceptual markings on freeways, the speed reduction and headway control performance of the markings might not be exactly the same as them have in the short-range application (or short time exposure). Because, essentially, we are still quite ignorant of how our visual perception would perform when we continuously and extensively exposed to visual cues of speed and distance within our visual field in a relatively open and high-speed scenario.

To this end, in this study, we proposed a kind of perceptual marking pattern specified to long-range application for the exit area of freeways, based on the previously well-verified reverse linear perspective markings. In this study, we name it as “enhanced reverse linear perspective markings (ERLPMs)”, which are supposed to address the abovementioned dilemma without sacificing the speed reduction and headway control effects. A detailed design of the ERLPMs will be introduced in the experimental design part as follows. To examine the effectiveness of the ERLPMs, a series of driving simulation experiments was conducted and the driving behavioral data were collected to test the performance of the noval ERLPMs on driving behavioral adjustment for accident prevention.

## 2 Methodology

**Fig 2:**
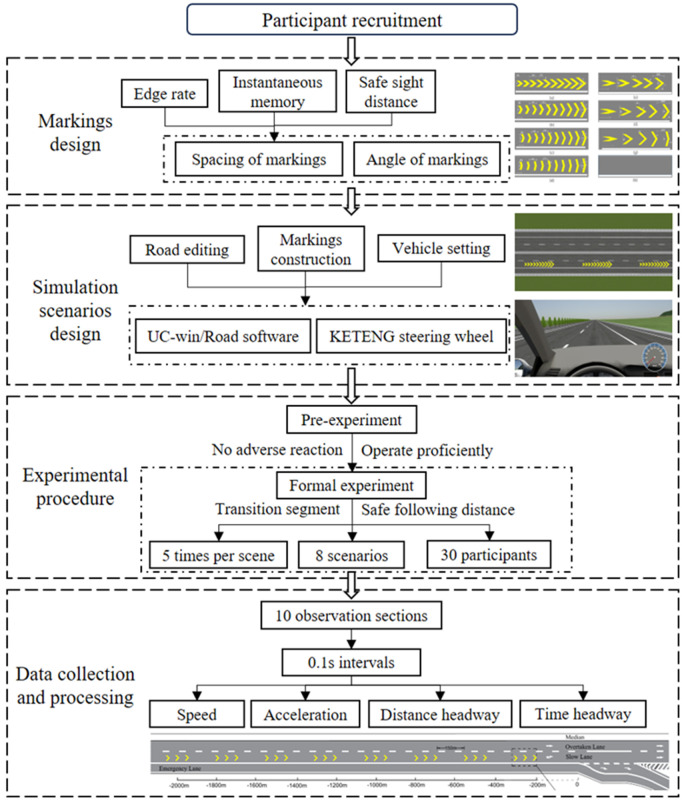
Framework of the driving simulation experiment and data collection.

In general, the driving simulation research paradigm was employed in this study. Wherein, a freeway scenario near the exit was simulated with various patterns of ERLPMs installed on road surface, and 30 participant drivers were recruited to driving in the above scenarios to collect their driving behavioral characteristics long the segment before and after the application of the perceptual markings. Fig 2 presents the framework of the entire driving simulation experiment and data collection.

### 2.1 Participants

In this experiment, the required sample size was examined and determined considering the expected variance, expected level of confidence, and margin of error, of the collected data sample, as shown in Equation (1):


$$N=\frac{Z^{\text{2}}\sigma^{\text{2}}}{E}$$ (1)

where N stands for the sample size; Z represents the standard normal distribution statistic; σ denotes the standard deviation; E represents the maximum error.

Typically, a 10% significance level was chosen to achieve a 90% confidence level for estimating unknown parameters [14]. When the confidence level is 90%, Z=1.25; the value of σ is 0.25-0.5, Due to limitations on the number of participants in the driving simulation experiments, σ and E were set to 0.4 and 10%, respectively. As a result, the required minimum sample size was 25. Considering the possibility of participants encountering difficulties in adapting to the driving simulator or other unexpected situations that could prevent them from completing the experiment, a total of 30 participants were recruited for this study. It was noticeable that all the recruited drivers were required to possess a valid driving license and had driving experience on freeways. The participants are mainly university students and we obtained their verbal consents of participation before the experiment under the witness of some other faculties of the university. The entire experiment starts on September 12, 2022 and ends on November 15, 2022.

Besides, the study only conducted the normal driving simulation experiments indoors, where the participants manipulated the steering wheel and accelerator or brake pedal while watching the road scenarios on the screen to simulate the driving process. And there were not any substantial risks to the physical or mental health of the participants. So, there is no need to obtain any ethics approval for this study.

### 2.3 Experimental design

#### 2.3.1 Enhanced reverse linear perspective markings (ERPLMs).

**Fig 3:**
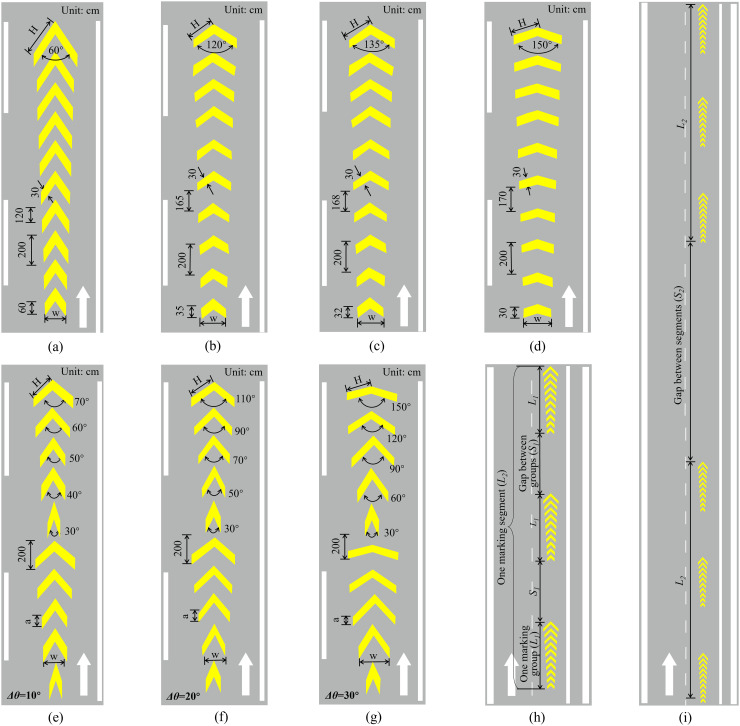
Designs and layouts of ERLPMs.


Based on the in introduction of the perceptual markings and the dilemma of long-range application of them, there two critical isuues neeVd to be addressed to expect to reach a better design of the markings, i.e., the enhanced reverse linear perspective markings (ERPLMs). The first one was to generate appropriate speed perception and distance perception at the same time; and second one was to find a specific deisgn to keep the effects of the perceptual markings on driving behaviors in a relatively long range yet without sacrificing the installation cost of them. According to the theories on linear perspective in visual perception [31,32] and previous field studies of RLPMs [19,43], the parameters of the reverse linear perspective markings (RLPMs) affect their effectiveness include length, angle, spacing, and size (see Fig 3). According to the previous studies, the angle of transverse pavement markings was recommended to be within the range of 30° to 150° (5, 7, 13), so the angle of the RPLMs in this study was designed to range from 30° to 150°. In order to address the issues concering the long-range application of the perceptual markings on freeways raised at the beginning, we specifically added two gaps within the markings series to form an intermittent visual stimuli and visual perception to avoid drviers’ visual adaptation and visual overload (see Figs 3(h), 3(i)). The first gap ($S_{\text{1}}$) was between individual marking groups, and the other gap ($S_{\text{2}}$) was between consecutive marking segments (see Figs 3(h), 3(i)). Wherein, the length of one marking group was set as 20 m, i.e., $L_{\text{1}}=\text{20}m$; and the length of one marking segment, consisting three consectutive marking groups, correspondingly equals to 100 m, i.e.s, $L_{\text{2}}=\text{20}m$. Besdies, the spacing of individual markings was determined based on the edge rate, which refers to the frequency that discontinuous texture of adjacent surfaces pass through a fixed reference point in the observer’s visual field. According to previous research [14,15], the maximum deceleration effect was observed when the edge rate equals 12 Hz. The edge rate could be given as Equation (2):

$$ER=\frac{V}{T_{X}}$$ (2)

where ER represents the edge rate (Hz), V denotes the velocity (m/s), and $T_{X}$represents the spacing of the texture (m). Since the running speed of the design scene in this paper was 23.6 m/s, the spacing was set to 2 meters.

The gap ($S_{\text{1}}$) between two consecutive marking groups was determined based on the drivers’ visual short-term memory (VSTM) [43,9,44]. VSTM is a type of memory system that is triggered by stimuli acting on sensory organs and typically lasts for about 1 second [45]. Under the condition of a running speed of 23.6 m/s, the distance within drivers’ VSTM is 23.6 m. To ensure that drivers can have effective visual perception of the perceptual markings within their VSTM distance, the gap between the marking groups were set as 20 m, i.e., $S_{\text{1}}=\text{20}m$ (see Fig 3(h)).

Aside from the distance within the drivers’ visual short-term memory for gaps between individual marking groups, an additional gap ($S_{\text{2}}$) between the consecutive marking segments was also considered. The additional gap ($S_{\text{2}}$) should be subjective to the drivers’ stopping sight distance (SSD) to ensure drivers’ uninterrupted visual perception from the markings. According to the Technical Standards of Highway Engineering [46], the stopping sight distance is given by Equation (3) as follows:


$$S=\frac{vt}{\text{3}.\text{6}}+\frac{v^{\text{2}}}{\text{2}\text{5}\text{4}(\phi +i)}+l$$ (3)

where S represents the stopping distance (m), v denotes the speed (m/s), t is the reaction time (s), ϕ is the coefficient of road surface friction, i represents the longitudinal slope of the road, and l is the safe distance (m). Specifically, according to Technical Standards of Highway Engineering, t is set to 2.5s, ϕ is set to 0.3 and i was set to 0 in this paper.

The safe distance l represents the minimum safe distance maintained between the vehicle and an obstacle after reaching to a complete stop. With the values of parameters mentioned above, S can be caculated as 153.8+l. Additionally, to ensure the drivers’ uninterrupted visual perception within the perceptual markings segment, a 150m-gap ($S_{\text{2}}=\text{150}m$) between two consecutive marking segments was chosen in this study.

In addition to the orginal RLPMs with extra gaps, the angle (θ) of a single marking (see Fig 2) of the RLPMs was particularly manipulated to expect to virtually enhance the ‘reverse’ effect of the RLPMs to generate enhanced effects on driving behavioral interventon [19,31,32,43]. That was, the fixed-angle pattern RLPMs and the variable-angle pattern RLPMs, which were all named as the ehanceed reverse linear perspective markings (ERLPMs). Specifically, the fixed angles (θ) were set as 60°, 120°, 135°, and 150°, respectively; and the variable angles (Δθ) were set as 10°, 20°, and 30°, respectively, starting from 30°. Besides, the orginal condition without any extra markings was treated as the baseline. Fig 3 depicts the design details and the long-rang layout of the ERPLMs on road surface. Wherein, the fixed-angle patterns are (a) θ=60°, (b) θ=120°, (c) θ=135°, and (d) θ=150°and the variable-angle patterns are (e) Δθ=10°, (f) Δθ=20°, and (g) Δθ=30°; Fig 3 (h) presents the layout of one ERLPMs segment; Fig 3 (i) shows the layout of two consecutive ERLPMs segments. Besdies, w represents the lateral width of a single marking, a denotes the vertical width of a single marking, H is the slash length, θ is the angle of a single marking, Δθ is the difference of two adjacent markings, $S_{\text{1}}$ is the gap between individual marking groups ($S_{\text{1}}=\text{20}m$), $S_{\text{2}}$ is the gap between two consecutive marking segments ($S_{\text{2}}=\text{150}m$), $L_{\text{1}}$ is the length of one marking group ($L_{\text{1}}=\text{20}m$), and $L_{\text{2}}$ is the length of one marking segment (consisting three consectutive marking groups, so $L_{\text{2}}=\text{100}m$).

#### 2.3.2 Simulation scenarios.

**Fig 4:**
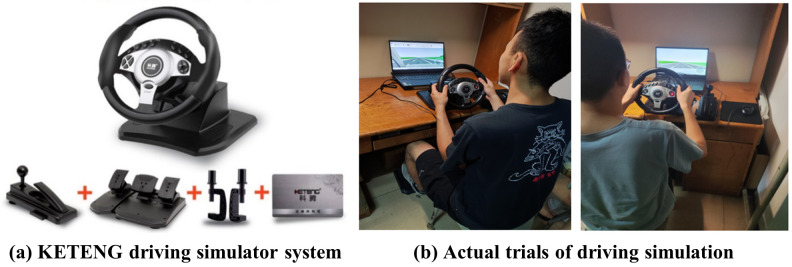
Driving simulator system and experiment.

The UC-win/Road software was used in this study to visualize the markings and the freeway segment, which is an effective tool for road design, traffic simulation, and control, enabling model manipulation such as scaling, moving, and rotation, and allowing configuration of driving parameters like speed and lane position. It supports simulation of traffic volumes and flow, and records driving indicators such as distance, speed, and acceleration. The experiment utilized a KETENG driving simulator system (see Fig 4), which includes a steering wheel, pedals, and a gear lever, mirroring the physical features of a real vehcile and providing a realistic driving experience.

The simulated scenario was designed to replicate the Guanshan Exit of an urban freeway in Wuhan. With a specific focus on the experimental goals of this study, the experimental traffic situation comprised a main vehicle followed by an experimental vehicle, creating a scenario of car-following status, while other lanes remained free from any additional vehicle interference. Subsequently, the ERLPMs was constructed and installed on the low-lane of the segment approaching an exit of the freeway (see Fig 4).

A passenger car was used as the vehicle model in the experiment. In the vehicle’s scenario, a target camera was created to simulate the driver’s perspective, adjusting the camera’s position to set its height at 1.3 meters, thus emulating the visual experience of the driver inside the car. As the main focus of this experiment was to investigate the impact of the ERLPMs on the driverss’ driving speed, acceleration, following distance and time headways, it was crucial to replicate real car-following conditions [47]. The experimental lane was situated on a freeway with a design speed of 100 km/h. In experiment, a preceding vehicle was set to travel at a speed of 90 km/h. To ensure that the participants can follow the preceding vehicle, a transition segment of 1 km was set in the upstream of the ERLPMs segment. During the transition segment, the participants were required to catch up with the preceding vehicle and maintain a self-perceived safe distance while following.

### 2.4 Procedures

Before the formal experiment, a 10-minute pre-experiment on another high-speed road scenario in the UC-win/Road system was conducted for each participant to familiarize themselves with the driving simulator. If discomfort or adverse conditions occur during practice, the participant would be excluded from the formal experiment, with unlimited practice attempts allowed until proficiency was achieved.

In the formal experiment, participants were instructed to follow specific guidelines: stay in the designated lane, avoid lane changes and speeding, maintain a safe following distance and prevent accidents. They were tasked to catch up with the vehicle ahead within a 1 km transition distance and maintain a self-perceived safe following distance. If they were failed to catch up with the leading vehicle as required, they will be forced to ceased immediately and be asked to restart the trial right away. The 8 scenarios were randomly arranged for each participant to complete their driving tasks (see Fig 4). And each participant conducted 5 repetitions per scenario with a break of 2-minutes between two consecutive trials, and a additional 10-minute rest was set between scenarios with varying ERPLMs.

### 2.5 Data collection and processing

Data collection was carried out using the LOG plugin embedded in the Uc-win/Road system. The LOG plugin automatically collects data such as vehicle travel time, distance, throttle/brake depth, position, vehicle speed, acceleration/deceleration, and lane width during the simulation process. The LOG plugin outputs data at regular time intervals, and for this experiment, the data output interval was set to 0.1 seconds. During the simulation process, the LOG plug-in (included with the UC-win/Road system) automatically collected data such as vehicle travel time, distance, throttle/brake depth, position, vehicle speed, acceleration and deceleration, and lane width and outputs them at 0.1s intervals.

In this experiment, the vehicle speed, acceleration, distance headway, and time headway were used for evaluate the performance of the ERLPMs on driving behavioral intervention. The driving data collected by the LOG plugin was processed and cleansed before its formal analysis.

**Fig 5:**
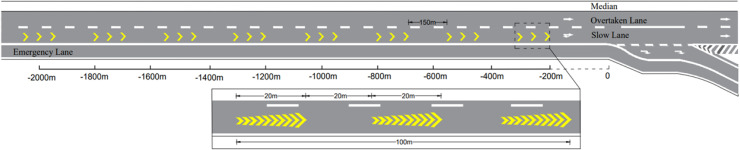
Illustration of the overall layout of the ERLPMs and the sectional data collection scheme.

Each experimental scenario included a 3-km segment with a 1-km transition at the beginning, and the driving behavioral data were collected every 200 meters within the 1.8 km from 2000 m away (labeled as observation section ‘-2000m’ in Fig 5) and 200 m away (labeled as observation section ‘-200m’ in Fig 4) from the starting section (labeled as ‘0’ in Fig 5) of the deceleration lane. Any abnormal data due to lane changes, accidents, and severe speeding, etc., were all removed. The vehicle speed, and acceleration, distance headway, and time headway at the ten observation sections (from ‘-2000m’ to ‘-200m’) were compared between the baseline and the conditions with varying ERLPMs.

In this study, vehicle speed, longitudinal acceleration, and longitudinal vehicle position data collected using the LOG plugin were utilized to calculate the experimental vehicle speed $v_{i}$, acceleration $a_{i}$, distance headway $d_{i}$, and time headway $h_{i}$, which were delivered by Equations (4)-(9) as follows:


$$d_{k}^{i}=x_{k}^{i}(\alpha )-x_{k}^{i}(\beta )$$ (4)

$$h_{k}^{i}=\frac{d_{k}^{i}}{v_{k}^{i}}$$ (5)

$$d_{i}=\frac{\sum_{k=\text{1}}^{n}d_{k}^{i}}{n}$$ (6)

$$h_{i}=\frac{\sum_{k=\text{1}}^{n}h_{k}^{i}}{n}$$ (7)

$$v_{i}=\frac{\sum_{k=\text{1}}^{n}v_{k}^{i}}{n}$$ (8)

$$a_{i}=\frac{\sum_{k=\text{1}}^{n}a_{k}^{i}}{n}$$ (9)

where x represents the longitudinal vehicle position, α denotes the leading vehicle, β refers to the experimental vehicle, i denotes the observation section, k represents the participant’s identification number, and n represents the experiment number.

### 2.6 Data analysis

An 8 (schemes: “Baseline”, “θ=60°”, “θ=120°”, “θ=135°”, “θ=150°”, “Δθ=10°”, “Δθ=20°”, “Δθ=30°”) × 10 (observation sections) ANOVA with repeated measures was employed to tested the effectiveness and difference of the ERPLMs on driving behavioral intervention. Post hoc comparisons were conducted using the Bonferroni test to adjusted p-values and minimized Type I errors, regardless of ANOVA results. These statistical analyses, including tests for normality and homogeneity of variance, were performed in RStudio (Version 20240.12.0) with R 4.4.2, at the 5% level of significance.

## 3 Results

### 3.1 Descriptive statistics of samples

There were 12000 (30 × 5×10 × 8) effective experiment data collected based on the above methods of data collection. The descriptive statistics and distributions of the final effective samples of all observations were summarized in Table 1. Besides, to facilitate the subsequent analysis of variance (ANOVA), Kolmogorov-Smirnov test and Levene’s test were conducted to examine the normality and homogeneity of variance of the collected data, respectively. The Kolmogorov-Smirnov test showed that all the p-values were less than 0.05 for speeds, distance headways, time headways, accelerations and lateral positions collected in varying observations, indicating non-normal distributions of the sampled data. The Levene’s test results also against the homogeneity of variance within varying observations and schemes regarding speed, distance headway, time headway, acceleration, and lateral position. Therefore, the paper utilized the alternative method of nonparametric test to conduct ANOVA (Scheirer-Ray-Hare) and multiple comparisons (Dunn’s Test with Bonferroni for p-value adjustment).

**Table 1:** Descriptive statistics of the final data samples.

ERLPMs	Speed (m/s)		Acceleration (m/s^2^)		Distance headway (m)		Time headway (s)		Lateral position (m)	
	Mean	SD	Mean	SD	Mean	SD	Mean	SD	Mean	SD
Baseline	24.76	1.19	-0.035	0.125	72.68	30.22	4.71	0.92	0.19	0.12
θ=60°	24.47	1.28	-0.030	0.114	78.75	29.61	5.04	0.82	0.20	0.13
θ=120°	24.41	1.28	-0.057	0.131	81.48	25.69	5.12	0.84	0.27	0.17
θ=135°	24.43	1.16	-0.044	0.122	79.51	27.52	4.98	0.87	0.25	0.15
θ=150°	24.51	1.27	-0.035	0.138	82.06	27.20	5.04	0.84	0.23	0.18
Δθ=10°	24.43	1.18	-0.041	0.106	83.41	27.84	4.75	0.81	0.27	0.20
Δθ=20°	24.44	1.21	-0.036	0.123	83.43	29.20	4.58	0.77	0.26	0.18
Δθ=30°	24.35	1.26	-0.045	0.119	78.17	27.54	4.61	0.68	0.23	0.17

Similar to the F-value in the ordinary parametric test, the H-value of the test statistic in the Scheirer-Ray-Hare results represents whether the difference between different schemes and observations on the dependent variable is significant, and the larger the H-value, the more significant the effect on the dependent variable. Besides, the significance level p was used to determine whether the H-value was significant. In addition, Dunn’s Test with Bonferroni was used for post hoc pairwise comparisons of the schemes and observations groups, and to determine whether the adjusted p-value met the condition to determine whether different schemes or different observations had a positive effect on the target factors (speed, distance headway, time headway, acceleration, and lateral position). It is important to note that a p-value of less than 0.05 shows a difference, while a p-value of less than 0.01 shows a significant difference.

### 3.2 Effects of ERLPMs on speed and acceleration

**Fig 6:**
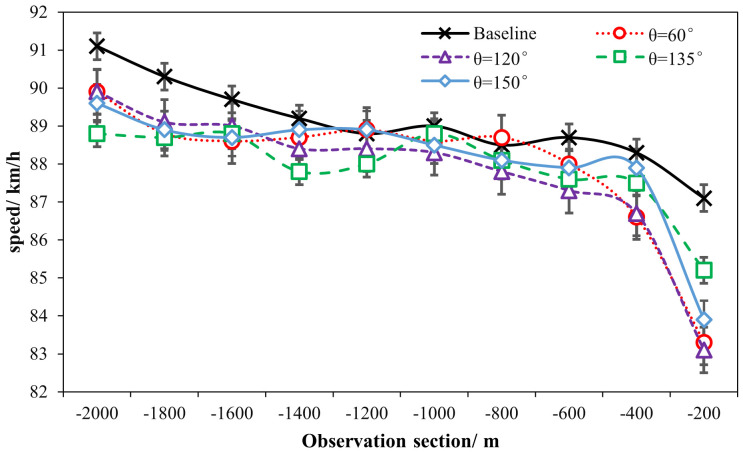
Sectional speeds under fixed-angle ERLPMs.

**Fig 7:**
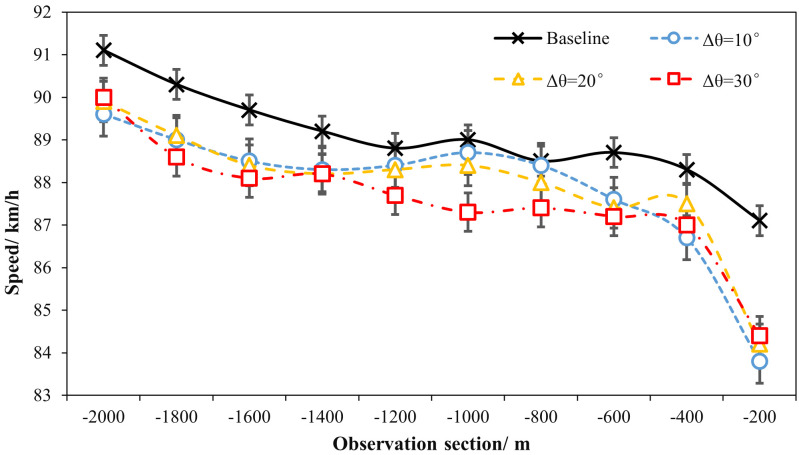
Sectional speeds under variable-angle ERLPMs.

**Fig 8:**
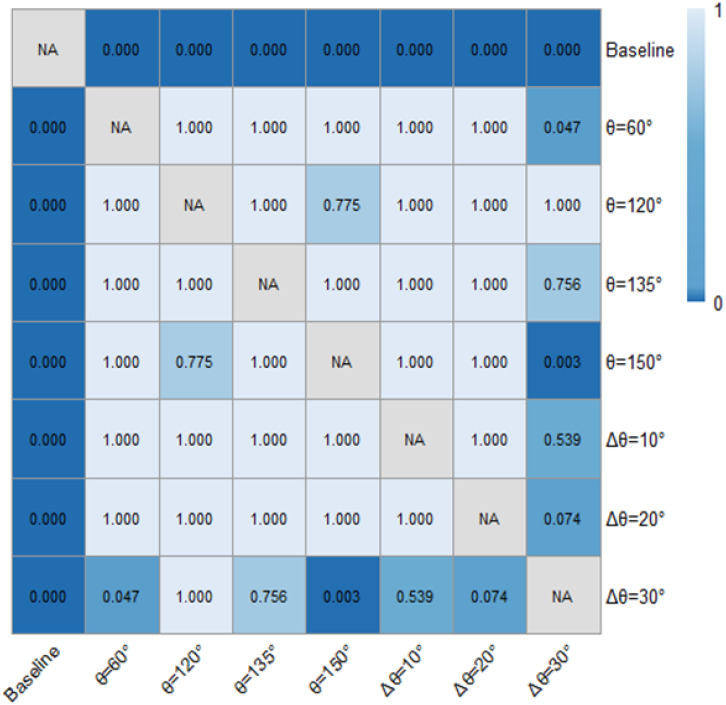
Post hoc pairwise comparisons of vehicle speeds of varying schemes.

**Fig 9:**
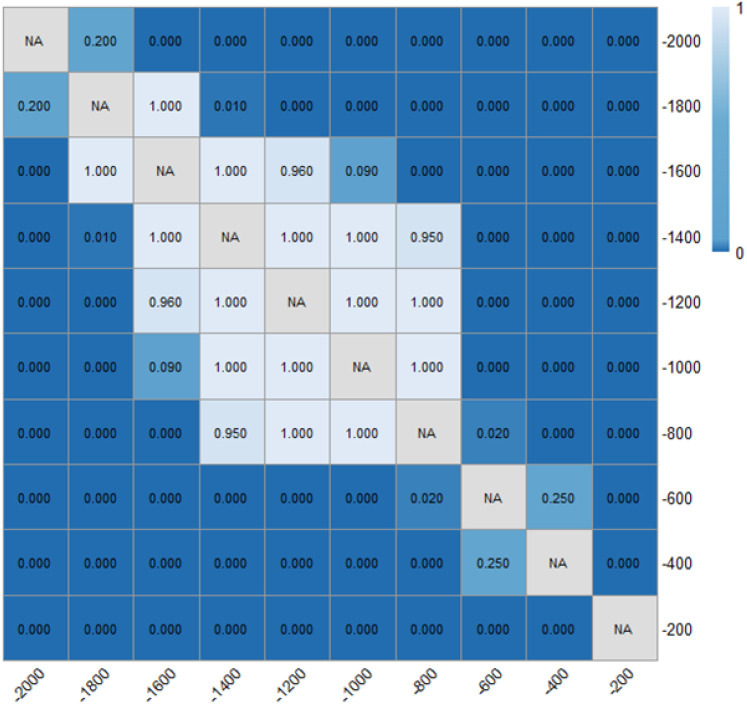
Post hoc pairwise comparisons of vehicle speeds of varying observation sections.

The comparisons of speed ($v_{i}$) and acceleration ($a_{i}$) among different observations and schemes were particularly reported here. Table 2 summarizes the ANOVA statistics of the effects of markings and observation sections on speed and acceleration. Fig 6 presents the sectional speeds under the conditions of fixed-angle pattern and variable-angle pattern of ERLPMs. The two-way ANOVA with eight schemes and ten observation sections revealed statistically significant main effects on speed for various schemes of ERLPMs (H = 111.135, p < 0.001), observation sections (H = 1298.523, p < 0.001), and their interaction (H = 97.019, p < 0.001). Particularly, as shown in Table 1, it can be discovered that, as compared with the baseline, the most significant speed reductions were produced by θ=120° (0.35 m/s) for the fixed-angle ERLPMs pattern and Δθ=30° (0.40 m/s) for the variable-angle ERLPMs pattern. Besides, according to Figs 6 and 7, it can be seen that after the installation of ERLPMs, the speed gradually reduced as the vehicle approaching the exit, and the greatest speed reductions from the initial observation section (‘-2000m’) to the final section (‘-200m’) were found to be 1.89 m/s with θ=120° and 1.61 m/s with Δθ=10°, respectively. And the greatest reductions in speed as above were all found to be extremely statistically significant as revealed by the post hoc pairwise comparisons as illustrated in Figs 8 and 9. Also, it could be noted that in all forms of the fixed- and variable-angle patterns of the ERLPMs, the speeds were founded to be significantly reduced as compared with the baseline (see Fig 8). Similarly, the speed at the final section (‘-200m’) was found to be significantly reduced as compared with any of the rest observation sections (Fig 9).

**Table 2:** Scheirer-Ray-Hare statistics of speed $v_{i}$ and acceleration $a_{i}$.

Statistics	Variable	Scheme	Observation section	Scheme × Observation section
Sum Sq	$v_{i}$	1.334 × 109	1.558 × 1010	1.164 × 109
	$a_{i}$	3.64 × 108	5.15 × 108	1.11 × 109
H-value	$v_{i}$	111.13542	1298.5226	97.019339
	$a_{i}$	30.350376	42.945539	92.814044
p-value	$v_{i}$	<0.001	<0.001	<0.001
	$a_{i}$	<0.001	<0.001	0.0086093

**Fig 10:**
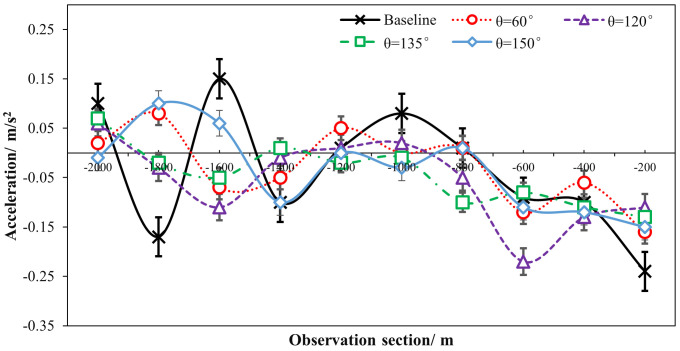
Sectional accelerations under fixed-angle ERLPMs.

**Fig 11:**
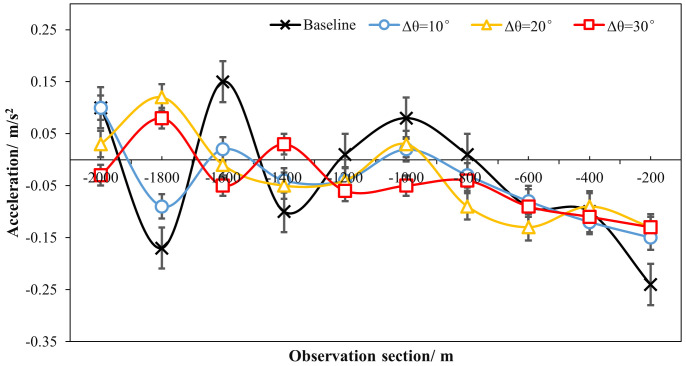
Sectional accelerations under variable-angle ERLPMs.

**Fig 12:**
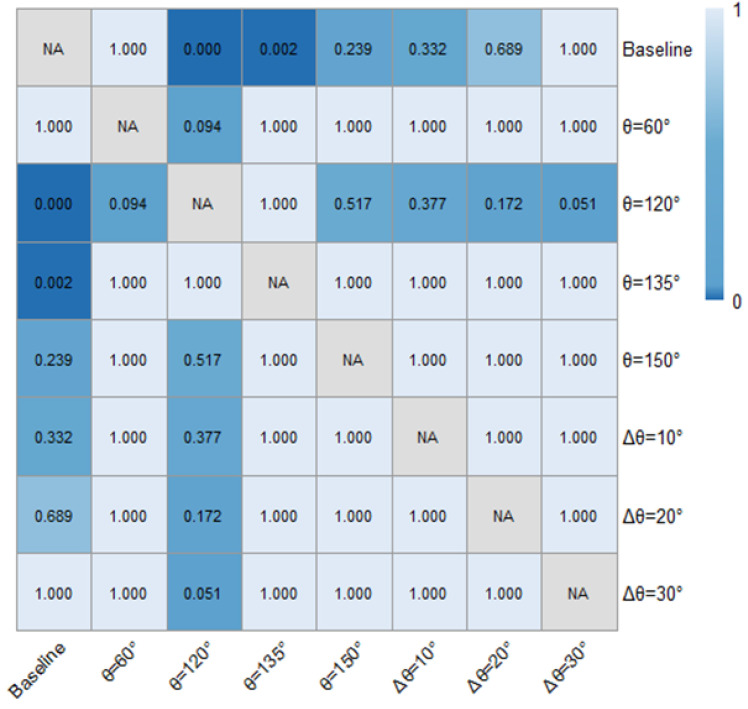
Post hoc pairwise comparisons of accelerations of varying schemes.

**Fig 13:**
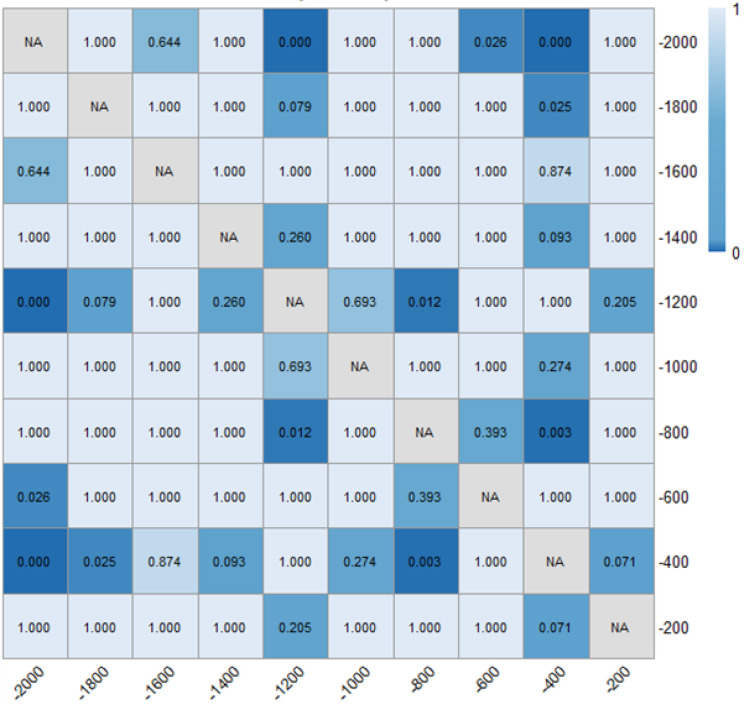
Post hoc pairwise comparisons of accelerations of varying observation sections.

As shown in Table 1 and Figs 10 and 11, the absolute accelerations were significantly decreased (a greater deceleration) when there were ERLPMs installed as compared with the baseline. Particularly, the greatest decreases in acceleration were found to be 0.22 m/s^2^ with θ=120° for the fixed-angle patterns and 0.010 m/s^2^ with Δθ=30° for the variable-angle ones. Also, generally, the acceleration decreased while approaching the exit, and the greatest decreases from observation sections ‘-2000m’ to ‘-200m’ were found in θ=120° (0.117 m/s^2^) for the fixed-angle pattern series and Δθ=10° (0.141 m/s^2^) for the variable-angle pattern series. Besides, a two two-way ANOVA showed that the main effects of ERLPMs (H=30.35, p < 0.001), observation sections (H = 42.95, p < 0.001), and their interaction (H = 92.81, p < 0.001) on acceleration were all found to be statistically significant. The post hoc pairwise comparisons revealed that only the comparisons between baseline and θ=120∘ (p < 0.001) and baseline and θ=135∘ (p < 0.01) were found to be statistically significant, yet the rest were not (see Fig 12). In addition, it can be discovered that in the baseline condition, the accelerations across observation sections oscilliated violently with positive and negative values (0.15 m/s^2^ and -0.24 m/s^2^ in maximum), especially during the first 1000 m (‘-2000m’ to ‘-1000m’), and have sudden acceleration drops (0.14 m/s^2^) at the last observation section. While in the ERLPMs conditions, the accelerations fluctuated at first 800m and then decreased continuously. Moreover, with both the fixed-angle patterns and the variable-angle patterns, the accelerations were significantly smoothed within the last 800m (‘-1000m’ to ‘-200m’) while approaching the exit when compared with the baseline condition (Fig 13). It means that the drviers effectively controlled their acceleration and speed while approaching the exit and avoided sudden brakes right before entering the exit.

### 3.3 Effects of ERLPMs on distance and time headways

**Fig 14:**
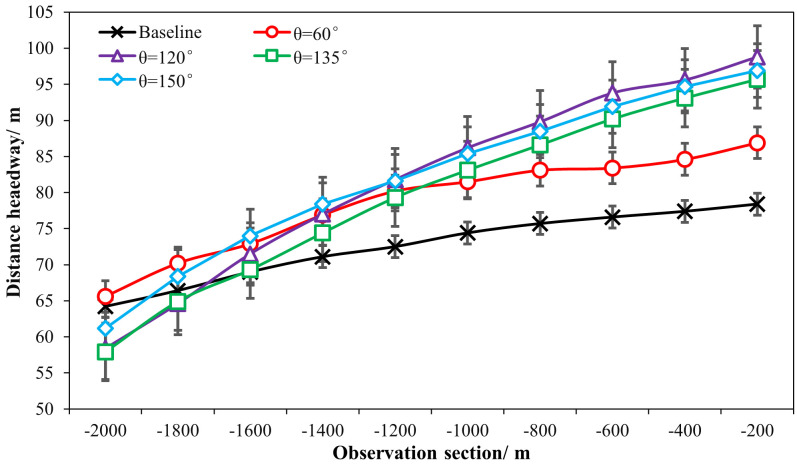
Sectional distance headways under fixed-angle ERLPMs.

**Fig 15:**
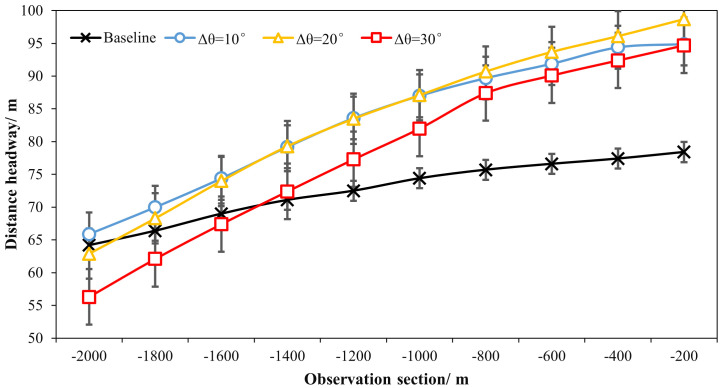
Sectional distance headways under changing-angle ERLPMs.

**Fig 16:**
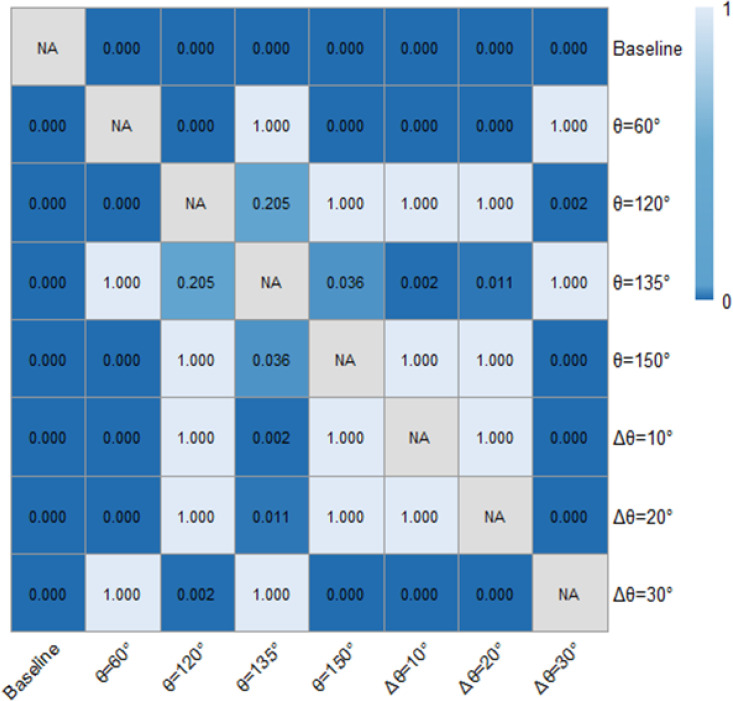
Post hoc pairwise comparisons of distance headways of varying schemes.

**Fig 17:**
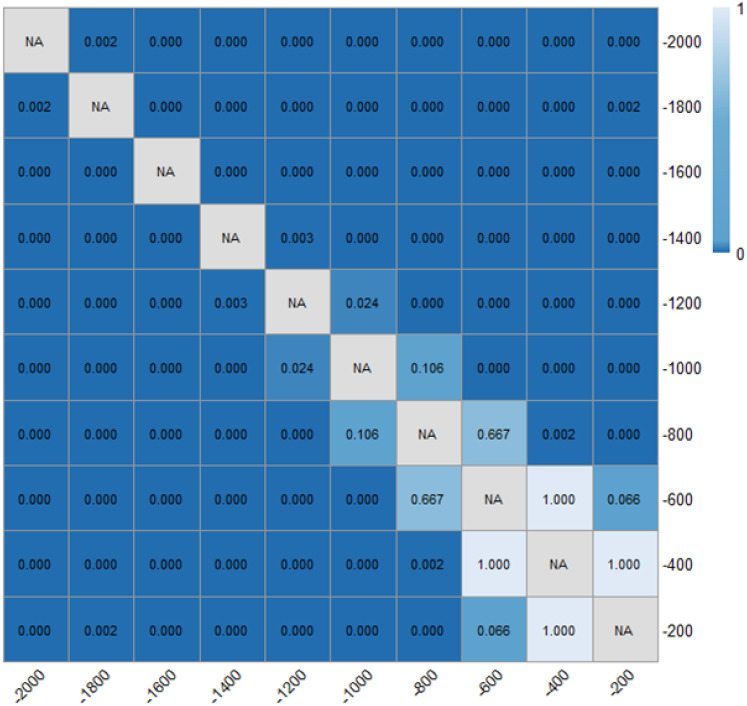
Post hoc pairwise comparisons of distance headways of varying observation sections.

As presented in Table 1 and Figs 14 and 15, it can be found that the distance headways ($d_{i}$) in varying fixed-angle patterns were all found to be increased as compared with the baseline, and the θ=120° pattern ERLPMs produced a 9.48 m increase in maxmum and the Δθ=30° pattern produced a 10.75 m increase in maxmum. Besides, according to Figs 14 and 15, it showed that the distance headway increased gradually from the intial observation section (‘-2000m’) to the last one (‘-200m’), and the largest increases were found in θ=120° (40.4 m) and Δθ=30° (38.4 m), which were significantly greater than that of the baseline (14.2 m). In addition, a two-way ANOVA revealed that the main effects of ERLPMs (H = 190.63, p < 0.001), observation sections (H = 1901.88, p < 0.001), and their interaction (H = 146.15, p < 0.001) were all found to be extremely significant (see Table 3). Further, as illustrated in Figs 16 and 17, the Dunn’s Test with Bonferroni for post hoc comparisons suggested that the pairwise comparisons of distance headway between the baseline condition and others with ERLPMs were all found to be statistically and significantly different. And the majority of the pairwise comparisons between the ERLPMs were also found to be significantly tested. As for the sectional differences of distance headway, the pairwise comparisons were all found to be statistically significant except for the comparisons between the distance headways at ‘-1000m’ and ‘-800m’ (p = 0.106), ‘-800m’ and ‘-600m’ (p = 0.667), ‘-600m’ and ‘-400m’ (p = 1.000), and ‘-400m’ and ‘-200m’ (p = 1.000).

**Table 3:** Scheirer-Ray-Hare statistics of distance headway $d_{i}$ and time headway $h_{i}$.

Statistics	Variable	Schemes	Observations	Schemes × Observations
Sum Sq	$d_{i}$	2.288 × 10^9^	2.282 × 10^10^	1.754 × 10^9^
	$h_{i}$	8,651 × 10^9^	6.066 × 10^10^	1.305 × 10^9^
H-value	$d_{i}$	190.62647	1901.8849	146.14834
	$h_{i}$	720.9594	5055.6749	108.74597
p-value	$d_{i}$	<0.001	<0.001	<0.001
	$h_{i}$	<0.001	<0.001	<0.001

**Fig 18:**
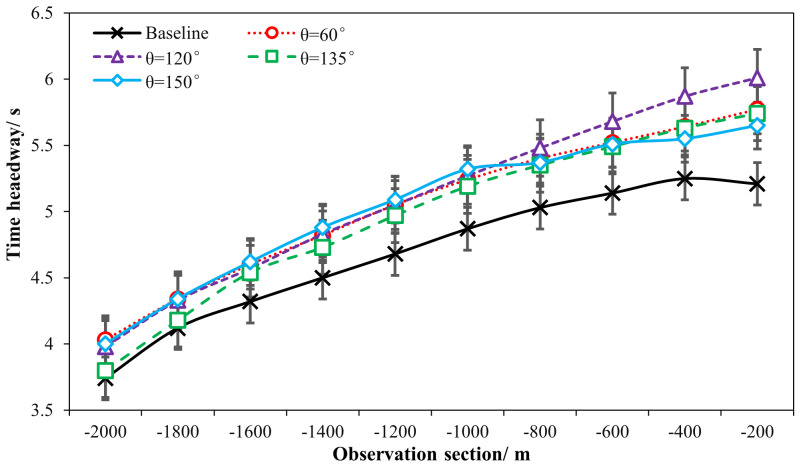
Sectional time headways under fixed-angle ERLPMs.

**Fig 19:**
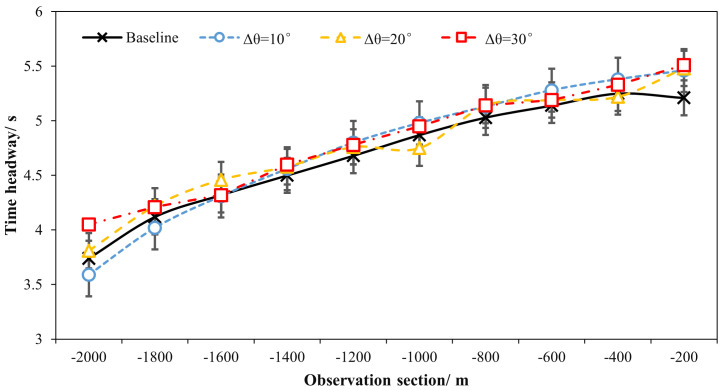
Sectional time headways under variable-angle ERLPMs.

**Fig 20:**
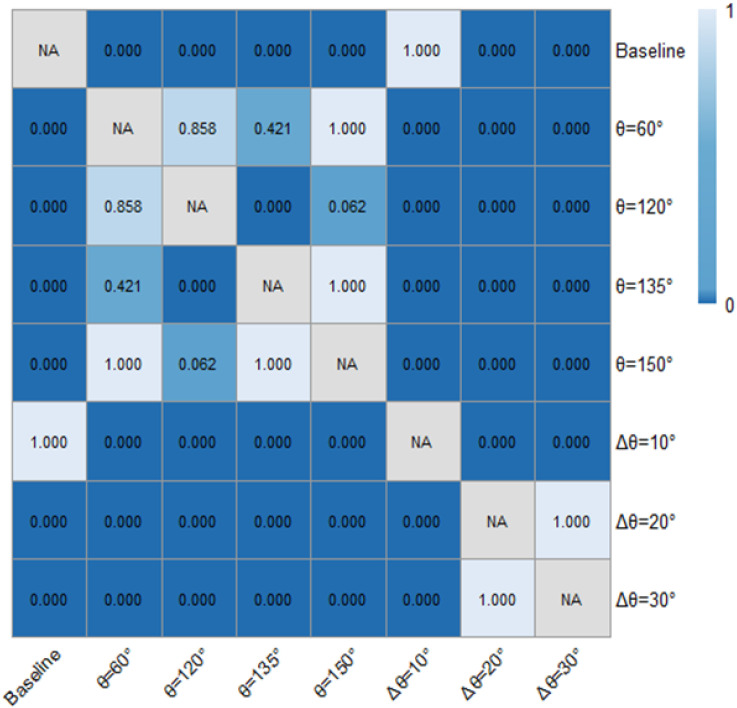
Post hoc pairwise comparisons of time headways of varying schemes.

**Fig 21:**
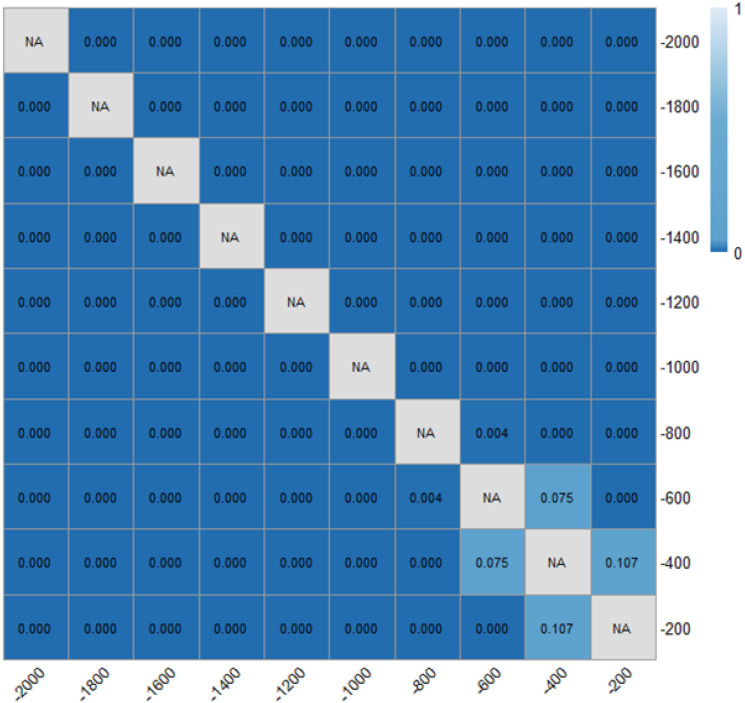
Post hoc pairwise comparisons of time headways of varying observation sections.

Table 1 and Figs 18 and 19 demonstrated the time heaway ($h_{i}$) variations with/ without the ERLPMs. It can be discovered that the fixed-angle patterns of ERLPMs unanimuously increased the time headway, and the largest increase (0.41s) was seen with the θ=120° pattern; yet the variable-angle patterns did not effectively lead to increase in time headway, with the largest increase of 0.04s with the Δθ=10° pattern. For the observation sectional variations, as illustrated in Figs 18 and 19, it can be seen that the greatest time headway increases were found in θ=120° pattern (2.03s) and Δθ=10° (1.87s), which were significantly greater than that of the baseline (1.47s). In addition, a two-way ANOVA revealed that the main effects of ERLPMs (H = 720.96, p < 0.001), observation sections (H = 5055.67, p < 0.001), and their interaction (H = 108.75, p < 0.001) were all found to be extremely significant. Further, as illustrated in Figs 20 and 21, the post hoc comparisons suggested that the pairwise comparisons of time headway between the baseline condition and others with ERLPMs were all found to be statistically and significantly different, except for the comparison between baseline and Δθ=10° (p = 1.000). And the majority of the pairwise comparisons between the ERLPMs were also found to be significant. As for the sectional differences of time headway, the pairwise comparisons were all found to be statistically significant expect for the comparisons between the time headwais at ‘-600m’ and ‘-400m’ (p = 0.075) and ‘-400m’ and ‘-200m’ (p = 0.107).

## 4 Discussion

### 4.1 Visually speed and distance perceptions manipulated by ERLPMs

The above results suggest that the ERLPMs were effective to induce drivers for a lower speed choice. This was in line with previous studies of perceptual markings revealing decreased speed after the application of the markings. Though the actual performance on speed reduction may vary from 0.278m/s to 2.78m/s as reported previously [7,23,24,13,14,19,15], the 1.89m/s reduction in this study could still be a strong support for choosing the ERLPMs even for speed reduction only, let alone for the long-range application as reported in the present study, which was revealed for the very first time. Specifically, it can be found in Figs 6–9 that the vehicle gradually and unanimously decreased while approaching the exit, expecially within the last 1000m, and with a smoothed acceleration variation. This, on the one hand, could be that the ERLPMs proposed in this study effectively affected the drivers’ speed perception due to the visual stimuli aroused by the specially designed markings [32,19,43]. On the other hand, it might as well suggest that the gaps ($S_{\text{1}}$ and $S_{\text{2}}$ in Figs 3(h), 3(i)) intentionally and specifically left between the marking groups and segments successfully led to an appropriate collective effect on drivers’ speed perception, which actually resulted in a substantial yet smoothed intervention of drivers’ speed choice with appropriate acceleration control.

Apparently, the headway control performance of the ERLPMs as above manifested our hypothesis of the extra function of the simple markings. This could be attribute to the fundamental yet critical theory of distance perception in cognitive psychology, which essentially holds that the distance can be underestimated if the predominant visual cues in the visual field are interrupted, isordered, or missing [20,21,40,31,32]. Specifically, as argured by [19,19], the reverse linear perspective markings effectively absorbed the advantages of the fundamental longitudinal perceptual markings and the various novel transverse ones. And it seemed that the fixed-angle pattern with θ=120°out performed others in both longitudinal and lateral driving behavioral intervention. It might be that the θ=120° pattern was more effectively recognized and perceived by drivers in terms of both speed and distance perception. Because the speed overestimation derives from the temporal frequency generated by the longitudinal visual stimulus with regard to the drivers’ moving direction, and the distance underestimation derives from ‘the discontinuity effects [42]’ and the ‘reverse’ effect of the markings [19,43,31,32], which subjective to how the original road surface visual texture was disrupted, especially in a transverse form with regard to the moving (visual searching) direction. Accordingly, the θ=120° pattern could be the best one in this study in terms of balancing the speed and distance perception and eventually producing the best outcome on driving behavior adjustment. Besides, comparatively, the variable-angle patterns may be a too much stimuli to drivers to easily and effectively read the visual cues in behind.

### 4.2 The balance between visual memory and visual pereption for long-range application

The dilemma of long-range application of perceptual markings essentially dwells in the balance between the cost-effectiveness nature of the markings and the sustained and appropriate visual perception (without over much visual load). So, left some gaps between marking groups intentionally could be a simple yet possibly effective way to implement the perceptual markings in a relative long range of road segment on freeways. But how to determin the specific gaps could be a fundamental issue in this regard, as it relates to the basic marking pattern design, and the drivers’ visual perception fluctuation, and visual load variations, and so on. In this study, the issue was innovatively and ingeniously addressed by introducing a dual-layer design strategy of the perceptual markings for their particular long-range applications. That was, the visual short-term memory (VSTM) and stopping sight distance (SSD) of drivers were embedded into the design of the perceptual marking patterns with the fundamental visual perception. In particular, the VSTM was used to determin the gap ($S_{\text{1}}$) between two consecutive marking groups [9,44,45], and the SSD was employed to determin the additional gap ($S_{\text{2}}$) between the consecutive marking segments (Ministry of Transport of the People’s Republic of China, 2014). Eventually, the VSTM ($S_{\text{1}}$) and SSD ($S_{\text{2}}$) combinedly shaped the perceptual markings in a long-range and the markings were verified to receive an encouraging driving behaivoral intervention performance to be the “ehnaced” version of RLPMs, i.e., ERLPMs. To our best knowledge, the ERLPMs proposed in this study were the first-of-its-kind solution for the long-range application of perceptual markings for accident prevention on freeways.

## 5 Conclusions

In this study, we proposed a kind of perceptual markings, i.e., the enhanced reverse linear perspective markings (ERLPMs), dedicated to the specific application in a long-range segment near the exit on freeways, and attempted to investigated their effectiveness on drivers’ speed, acceleration, headway controls in car-following situations. This was achieved by conducting a series of driving simulation experiments with seven forms of the ERLPMs installed on the road surface, wherein four were categorized into the fixed-angle pattern and the rest three belonged to the variable-angle pattern, which were all designed according to the effects of reverse linear perspective visual cues on speed and distance perception. Twenty-five participants involved in the experiment, and speed, acceleration, distance and time headways were collected. The findings of this study can be concluded as follows:

1) the ERLPMs could effectively lead to substantial reduction of speed and increase in headways while vehiles approaching the exits in car-following states during a long-range segment;
2) the greatest speed reduction, acceleration reduction, distance headway increase, and time headway increase were 0.40 m/s, 0.22 m/s^2^, 10.75 m, and 0.41 s, respecptively, as compared with the baseline (no extra markings);
3) the fixed-angle pattern with θ=120° had the most impressive and stabilized performance on all the speed and headway control aspects;
4) the ERLPMs were capable of adjusting driver behaviors quite smoothly, which is especially beneficial for the accident prevention at exit area of freeways.

However, some limitations remain in that, 1) although the dirivng simulation experiment is convenient for testing the proposed novel forms of perceptual markings, the ERPLMs still needs to be verified on real-world roadways for its practical usage and modification; 2) the traffic volume, traffic composition, drivers’ personal characteristics, and some other factors may also have a impact on the performance of the markings; 3) also, there still could be some other forms of perceptual markings to further ‘enhance’ the performance on driving behavior intervention and crash risk mitigation, and the position, layout, and color, etc., could all be specific factors that need to be considered in the future optimization of the markings; 4) aside from the exit area on freeways, some other accident-prone sites, such as the tangent segment linking the curves with small radius, the tunnels, long downhill segments, and so on, are also worth of application of the ERLPMs, but the design, overall layout, and final performance of them may vary greatly as compared to the exit area of freeways. The above limitations were not supposed to be covered in the present study, but are within our future research directions.

## References

[1] National Center for Statistics and Analysis. Traffic safety facts 2022: A compilation of motor vehicle traffic crash data. DOT HS 813 656. Washington, D.C.: National Highway Traffic Safety Administration; 2024.

[2] Traffic Management Bureau. Annual statistics of road traffic accidents of People’s Republic of China (2020). Traffic Management Bureau, The Ministry of Public Security of the People’s Republic of China, Beijing. (in Chinese); 2021.

[3] Charlton SG. The role of attention in horizontal curves: a comparison of advance warning, delineation, and road marking treatments. Accid Anal Prev. 2007;39(5):873–85. doi: 10.1016/j.aap.2006.12.007 17217906

[4] Charlton SG, Starkey NJ. Risk in our midst: Centrelines, perceived risk, and speed choice. Accid Anal Prev. 2016;95(Pt A):192–201. doi: 10.1016/j.aap.2016.07.019 27450791

[5] Charlton SG, Starkey NJ, Malhotra N. Using road markings as a continuous cue for speed choice. Accid Anal Prev. 2018;117:288–97. doi: 10.1016/j.aap.2018.04.029 29751138

[6] Coutton-Jean C, Mestre DR, Goulon C, Bootsma RJ. The role of edge lines in curve driving. Transportation Research Part F: Traffic Psychology and Behaviour. 2009;12(6):483–93. doi: 10.1016/j.trf.2009.04.006

[7] Montella A, Aria M, D’Ambrosio A, Galante F, Mauriello F, Pernetti M. Perceptual Measures to Influence Operating Speeds and Reduce Crashes at Rural Intersections: Driving Simulator Experiment. Transportation Research Record: Journal of the Transportation Research Board. 2010;2149(1):11–20. doi: 10.3141/2149-02

[8] Montella A, Aria M, D’Ambrosio A, Galante F, Mauriello F, Pernetti M. Simulator evaluation of drivers’ speed, deceleration and lateral position at rural intersections in relation to different perceptual cues. Accid Anal Prev. 2011;43(6):2072–84. doi: 10.1016/j.aap.2011.05.030 21819837

[9] Zhang H, Hou N, Ding N, Jiao N. Using multicolor perceptual markings as a rear-end crash risk mitigator: A field investigation. Accid Anal Prev. 2023;179:106881. doi: 10.1016/j.aap.2022.106881 36327679

[10] Zhao X, Ding H, Lin Z, Ma J, Rong J. Effects of longitudinal speed reduction markings on left-turn direct connectors. Accid Anal Prev. 2018;115:41–52. doi: 10.1016/j.aap.2018.02.027 29544136

[11] Zhao X, Ju Y, Li H, Zhang C, Ma J. Safety of Raised Pavement Markers in Freeway Tunnels Based on Driving Behavior. Accid Anal Prev. 2020;145:105708. doi: 10.1016/j.aap.2020.105708 32781174

[12] Calvi A. Investigating the effectiveness of perceptual treatments on a crest vertical curve: A driving simulator study. Transportation Research Part F: Traffic Psychology and Behaviour. 2018;58:1074–86. doi: 10.1016/j.trf.2018.06.002

[13] Wood J, Donnell ET. Empirical Bayes before-after evaluation of horizontal curve warning pavement markings on two-lane rural highways in Pennsylvania. Accid Anal Prev. 2020;146:105734. doi: 10.1016/j.aap.2020.105734 32827844

[14] Ding N, Jiao N, Zhu S, Liu B. Structural equations modeling of real-time crash risk variation in car-following incorporating visual perceptual, vehicular, and roadway factors. Accid Anal Prev. 2019;133:105298. doi: 10.1016/j.aap.2019.105298 31557617

[15] Ding N, Zhu S, Jiao N, Liu B. Effects of peripheral transverse line markings on drivers’ speed and headway choice and crash risk in car-following: A naturalistic observation study. Accid Anal Prev. 2020;146:105701. doi: 10.1016/j.aap.2020.105701 32823033

[16] Engström J, Johansson E, Östlund J. Effects of visual and cognitive load in real and simulated motorway driving. Transportation Research Part F: Traffic Psychology and Behaviour. 2005;8(2):97–120. doi: 10.1016/j.trf.2005.04.012

[17] Han L, Du Z, He S, Wang S. An empirical investigation of driver’s eye-catching effect in the entrance zone of freeway tunnels: A naturalistic driving experiment. Transportation Research Part F: Traffic Psychology and Behaviour. 2024;101:92–110. doi: 10.1016/j.trf.2024.01.004

[18] Wang S, Zheng H, Du Z, Han L, He S, Jiao F. Comprehensive Evaluation of Visual Guiding Systems for Enhancing Traffic Safety in Freeway Tunnels: An Improved Matter-Element Method with Case Study. Transportation Research Record: Journal of the Transportation Research Board. 2024;2678(10):957–71. doi: 10.1177/03611981241236185

[19] Ding N, Zhu S, Wang H, Jiao N. Effects of reverse linear perspective of transverse line markings on car-following headway: A naturalistic driving study. Safety Science. 2019;119:50–7. doi: 10.1016/j.ssci.2018.08.021

[20] Gibson JJ. The perception of the visual world. Boston: Houghton Mifflin. 1950.

[21] Warren R. Optical transformation during movement: Review of the optical concomitants of egomotion. AD-A122275. Columbus, OH: Ohio State University, Department of Psychology. 1982.

[22] Ding N, Jiao N. Long-term effectiveness of reverse linear perspective markings on crash mitigation in car-following: Evidence from naturalistic observations. Accid Anal Prev. 2021;159:106273. doi: 10.1016/j.aap.2021.106273 34218196

[23] Hunter M, Boonsiripant S, Guin A, Rodgers MO, Jared D. Evaluation of Effectiveness of Converging Chevron Pavement Markings in Reducing Speed on Freeway Ramps. Transportation Research Record: Journal of the Transportation Research Board. 2010;2149(1):50–8. doi: 10.3141/2149-06

[24] Hunter M, Rodgers MO, Pratyaksa P. Safety performance evaluation of converging chevron pavement markings. Georgia Department of Transportation, Office of Materials & Research; 2014.

[25] Martindale A, Urlich C. Effectiveness of transverse road markings on reducing vehicle speeds. 423. Wellington: NZ Transport Agency. 2010.

[26] Akbari A, Haghighi F. Traffic calming measures: An evaluation of four low-cost TCMs’ effect on driving speed and lateral distance. IATSS Research. 2020;44(1):67–74. doi: 10.1016/j.iatssr.2019.07.002

[27] Du Z, Deng M, Lyu N, Wang Y. A review of road safety evaluation methods based on driving behavior. Journal of Traffic and Transportation Engineering (English Edition). 2023;10(5):743–61. doi: 10.1016/j.jtte.2023.07.005

[28] Bei R, Du Z, Lyu N, Yu L, Yang Y. Exploring the Mechanism for Increased Risk in Freeway Tunnel Approach Zones: A Perspective on Temporal-spatial Evolution of Driving Predictions, Tasks, and Behaviors. Accid Anal Prev. 2025;211:107914. doi: 10.1016/j.aap.2024.107914 39787825

[29] Zong F, Wang S-Q, Qin Y-Z, Zeng M. Analyzing the short- and long-term car-following behavior in multiple factor coupled scenarios. Expert Systems with Applications. 2025;272:126724. doi: 10.1016/j.eswa.2025.126724

[30] Tarko AP, Davis GA, Saunier N, Sayed T. White paper: surrogate measures of safety. Washington, D.C. 2009.

[31] Wu B, Ooi TL, He ZJ. Perceiving distance accurately by a directional process of integrating ground information. Nature. 2004;428(6978):73–7. doi: 10.1038/nature02350 14999282

[32] Wu B, He ZJ, Ooi TL. The linear perspective information in ground surface representation and distance judgment. Percept Psychophys. 2007;69(5):654–72. doi: 10.3758/bf03193769 17929690

[33] Ding H, Zhao X, Rong J, Ma J. Experimental research on the effectiveness of speed reduction markings based on driving simulation: a case study. Accid Anal Prev. 2013;60:211–8. doi: 10.1016/j.aap.2013.08.007 24077218

[34] Ding H, Zhao X, Rong J, Ma J. Experimental research on the effectiveness and adaptability of speed reduction markings in downhill sections on urban roads: a driving simulation study. Accid Anal Prev. 2015;75:119–27. doi: 10.1016/j.aap.2014.11.018 25460098

[35] Awan HH, Pirdavani A, Houben A, Westhof S, Adnan M, Brijs T. Impact of perceptual countermeasures on driving behavior at curves using driving simulator. Traffic Inj Prev. 2019;20(1):93–9. doi: 10.1080/15389588.2018.1532568 30822137

[36] Babić D, Stjepan Žebec M, Babić D, Čavka M. Effect of chevron design on driver behaviour when encountering and passing through a dangerous curve. Transportation Research Part F: Traffic Psychology and Behaviour. 2022;86:370–83. doi: 10.1016/j.trf.2022.03.010

[37] Garach L, Calvo F, De Oña J. The effect of widening longitudinal road markings on driving speed perception. Transportation Research Part F: Traffic Psychology and Behaviour. 2022;88:141–54. doi: 10.1016/j.trf.2022.05.021

[38] Ding N, Zhu S, Wang H, Jiao N. Effects of edge rate of the designed line markings on the following time headway. Scientia Iranica. 2017;24(4):1770–8. doi: 10.24200/sci.2017.4268

[39] Larish JF, Flach JM. Sources of optical information useful for perception of speed of rectilinear self-motion. J Exp Psychol Hum Percept Perform. 1990;16(2):295–302. doi: 10.1037//0096-1523.16.2.295 2142200

[40] Sinai MJ, Ooi TL, He ZJ. Terrain influences the accurate judgement of distance. Nature. 1998;395(6701):497–500. doi: 10.1038/26747 9774104

[41] Yarbrough GL, Wu B, Wu J, J. He Z, Leng T. Judgments of object location behind an obstacle depend on the particular information selected. Journal of Vision. 2010;2(7):625–625. doi: 10.1167/2.7.625

[42] Feria CS, Braunstein ML, Andersen GJ. Judging distance across texture discontinuities. Perception. 2003;32(12):1423–40. doi: 10.1068/p5019 14992421

[43] Ding N, Lu Z, Jiao N, Liu Z, Lu L. Quantifying effects of reverse linear perspective as a visual cue on vehicle and platoon crash risk variations in car-following using path analysis. Accid Anal Prev. 2021;159:106215. doi: 10.1016/j.aap.2021.106215 34130057

[44] Du J, Ren G, Liu W, Li H. How is the visual working memory load of driver influenced by information density of traffic signs?. Transportation Research Part F: Traffic Psychology and Behaviour. 2022;86:65–83. doi: 10.1016/j.trf.2022.02.007

[45] Chapman P, Orhan S, Moore L. Short term memory and peripheral vision at junctions. Transportation Research Part F: Traffic Psychology and Behaviour. 2023;95:432–49. doi: 10.1016/j.trf.2023.05.004

[46] Ministry of Transport of the People’s Republic of China. Technical Standards of Highway Engineering (JTG B01-2014). Beijing: Beijing. 2014.

[47] Zong F, Yue S, Zeng M, Liu Y, Tang J. Environment reconstruction and trajectory planning for automated vehicles driving through signal intersection. Physica A: Statistical Mechanics and its Applications. 2025;660:130323. doi: 10.1016/j.physa.2024.130323

